# Clinical significance of mismatch repair gene expression in sporadic colorectal cancer

**DOI:** 10.3892/etm.2014.1927

**Published:** 2014-08-22

**Authors:** ZHENQIANG SUN, XIANBO YU, HAIJIANG WANG, SHUO ZHANG, ZELIANG ZHAO, RUIWEI XU

**Affiliations:** 1 Department of Gastrointestinal Surgery, Affiliated Tumor Hospital, Xinjiang Medical University, Urumqi, Xinjiang 830011, P.R. China; 2 Research Laboratory of Disease Genomics, Cancer Research Institute, Central South University, Changsha, Hunan 4170078, P.R. China; 3 Department of Pathology, The Second Affiliated Hospital, Xinjiang Medical University, Urumqi, Xinjiang 830011, P.R. China; 4 Infection & Statistical Office, Affiliated Tumor Hospital, Xinjiang Medical University, Urumqi, Xinjiang 830011, P.R. China

**Keywords:** mismatch repair genes, sporadic colorectal cancer, clinicopathological parameters, immunohistochemistry, prognosis

## Abstract

Mismatch repair (MMR) genes play an important role in the occurrence and development of sporadic colorectal cancer; however, the effect of MMR genes on clinicopathological features and prognosis remains unclear. The aim of the present study was to observe the clinical significance of MMR gene expression in sporadic colorectal cancer. Clinicopathological data and postoperative samples from 404 patients with sporadic colorectal cancer were obtained from the Affiliated Tumor Hospital of Xinjiang Medical University. The immunohistochemistry PV-9000 two-step method was performed to measure the protein expression of human mutL homolog 1 (hMLH1), human mutS homolog (hMSH) 2, human postmeiotic segregation increased 2 (hPSM2) and hMSH6. Differences in clinicopathological features, family history and survival time subsequent to surgery between groups with normal and aberrant MMR protein (MMRP) expression were compared. A total of 27.23% of all patients showed aberrant nuclear staining of MMRP. Among the patients with aberrant MMRP expression, a higher proportion of patients showed aberrant expression of more than one type of MMRP than aberrant expression of only one type of MMRP. Aberrant expression of hMLH1/hPSM2 was most commonly observed (29/404). In addition, aberrant MMRP expression in colorectal cancer was indicated predominantly in the right hemicolon. Histological type primarily showed mucinous adenocarcinoma. In addition, with increasing body mass index (BMI), the MMRP deficiency rate was also shown to increase gradually. There was a close association between MMRP expression deficiency and family history of cancer (P<0.05). For TNM stage III patients, the Kaplan-Meier survival curve showed that the aberrant MMRP expression group had a three-year disease-free survival (DFS) rate of 66.67%, which was longer than the DFS rate of the normal group (55.41%), with no statistical difference (P>0.05). In conclusion, the immunohistochemistry PV-9000 two-step method can be used to measure MMRP expression in colorectal cancer. Aberrant MMRP expression is closely correlated with tumor location, histological type, BMI and tumor family history in sporadic colorectal cancer. Aberrant MMRP expression may have an effect on the prognosis of stage III patients.

## Introduction

The occurrence and development of colorectal cancer is a complicated multi-step process, which involves numerous factors and genes. A number of tumor-related events are involved in this process, including oncogene activation, tumor suppressor gene inactivation, mismatch repair (MMR) gene mutations and gene promoter hypermethylation (1,2). Since the identification of MMR genes, studies have investigated the association between the aberrant expression of MMR genes and hereditary nonpolyposis colorectal cancer (HNPCC) or sporadic colorectal cancer (3–5). A number of studies have found that aberrant MMR gene expression plays an important role in the occurrence of colorectal cancer (6,7). At present, numerous genes are known to be involved in the MMR process, including human mutL homolog 1 (hMLH1), human mutS homolog (hMSH) 2, hMSH6, human postmeiotic segregation increased (hPSM) 1, hPSM2, hMSH3 and hMSH5. The protein products of MMR expression are enzymes that can repair mismatched base groups in the DNA replication process in order to maintain the fidelity of DNA replication.

At present, there are numerous studies investigating the pathogenesis of HNPCC (8,9); however, fewer studies have investigated the role of MMR gene mutations in sporadic colorectal cancer and microsatellite instability (MSI). A previous study found that ~15% of sporadic colorectal cancer cases exhibit a similar pathogenesis to HNPCC (10). However, the contribution of MMR gene mutation to the pathogenesis of these two types of colorectal cancer is considered to be different.

It has been indicated in a number of previous studies (11,12) that MMR gene mutations can result in tumorigenesis through two mechanisms. Firstly, simple sequence repeats can cause homologous genetic recombination in the DNA replication process. Consequently, variations in the sequence containing the simple sequence repeats increase DNA MSI in tumor cells. Secondly, aberrant MMR gene expression can result in the accelerated accumulation of gene mutations in proto-oncogenes and cancer suppressor genes. Consequently, this can affect the proliferation regulation of normal cells. In recent years, studies have focused on four types of MMR genes, MLH1, MSH2, MSH6 and PSM2 (13–15).

In excess of 90% of patients with HNPCC have DNA with high MSI (MSI-H), suggesting that the occurrence of HNPCC is associated with the functional loss of cell MMR. Therefore, DNA MSI can be regarded as a reliable indicator to measure the function of cell MMR (16). A typical characteristic of MMR gene mutation is MSI expression. The microsatellite sequence mutation rate due to MMR of tumor cells is 100–1,000 fold higher than that of normal cells. Furthermore, the MSI in colorectal tumors caused by aberrant MMR gene expression is ~15% (17). Therefore, detecting MSI is of high value. MSI may be used as a positive prognostic factor for sporadic colorectal cancer (18), but also as a negative forecasting sign for fluorouracil (5-FU)-based chemotherapy (19,20).

At present, there are there are few studies investigating MMR genes in sporadic colorectal cancer. Therefore, in the present study, clinicopathological data and 404 postoperative samples from patients with sporadic colorectal cancer were collected from the Tumor Hospital of Xinjiang Medical University (Urumqi, China). The aims of the study were to detect MMR protein (MMRP) expression using immunohistochemistry, in order to elucidate how aberrant MMRP expression was distributed in Chinese patients with sporadic colorectal cancer, and to analyze the association between aberrant MMRP expression and clinicopathological features, in order to investigate their prognostic effect.

## Materials and methods

### Patients and clinicopathological parameters

Clinicopathological data and postoperative samples from 404 patients with sporadic colorectal cancer were collected between May 2009 and June 2012 from the Tumor Hospital of Xinjiang Medical University. Parameters involved age at diagnosis, gender, nationality, body mass index (BMI) at diagnosis, anemia, tumor size, histological type, degree of differentiation, general type, TNM stage, tumor location, family history of cancer and histopathology report (shown in Table I). The diagnostic criteria were as follows: Anemia, male hemoglobin (Hb) <120 g/l or female Hb <115 g/l; BMI, lean <18.5 kg/m^2^, normal 18.5–23.9 kg/m^2^, overweight 24–27.9 kg/m^2^ and obese ≥28 kg/m^2^; HNPCC, diagnosis according to the Amsterdam II criteria (21). Cases were excluded due to HNPCC diagnosis, preoperative chemoradiotherapy or lack of data. Informed consent was obtained from all the patients prior to inclusion in the study and this study was approved by the Medical Ethics Committee of the Affiliated Tumor Hospital of Xinjiang Medical University (no. W201302).

### Immunohistochemistry

The neutral formalin-fixed (concentration, 40 g/l), paraffin-embedded specimens were serially sectioned (5-μm thickness), and a PV-9000 two-step method was performed using mouse anti-human monoclonal antibodies against MLH1, MSH2, MSH6 and PSM2 (Beijing Zhongshan Golden Bridge Biotechnology Co., Ltd., Beijing, China) as primary antibodies with a working concentration of 1:150. A universal two-step method (horseradish peroxidase) detection kit (Fujian Maixin Biological Products Co., Ltd., Fuzhou, China) was utilized. Phosphate-buffered saline was used instead of primary antibodies as a negative control, while normal colorectal mucosa and/or infiltrating lymphocytes were used as a positive control. Positive expression of MLH1 and MSH2 was observed in the nucleus. The results were analyzed in accordance with the method previously described by Plevová *et al* (22), in which the number of microscopic tumor cells showing positive nuclear staining was combined with the staining intensity and percentage of positive cells to determine the positive expression levels. A total of five high-power fields were selected from each sample using a light microscope and 100 cells were counted in each field. The grading of staining intensity was as follows: no staining, 0 points; light yellow, 1 point; yellow, 2 points; and brown, 3 points. The grading of the percentage of positive cells was as follows: No positive cells, 0 points; ≤10%, 1 point; 11–50%, 2 points; 51–75%, 3 points; and >75%, 4 points. If the result obtained by multiplying the two scores above was ≥2 points, the case was considered to have positive expression; however, if the score was <2 points, the case was considered to have negative expression. The positive control was positive nuclei of normal colorectal mucosa and/or infiltrating lymphocytes. However, a negative result was judged in the case of positive nuclear expression in the positive control and missing staining in the tumor cell nuclei.

### Statistical analysis

Univariate analysis was performed using the χ^2^ test. Multivariate correlation analysis was performed using the logistic regression test. Disease-free survival (DFS) was analyzed using the Kaplan-Meier method and the Log-rank test was used for comparison between groups. The statistical analysis was performed using SPSS for Windows version 18 (SPSS Inc., Chicago, IL, USA). The Fisher’s exact test from the statistical package STATA 9.0 (Stata Corp., College Station, TX, USA) was used for the calculations. P<0.05 was considered to indicate a statistically significant difference.

## Results

### Immunohistochemical results of MMRP expression

**Figure 1:**
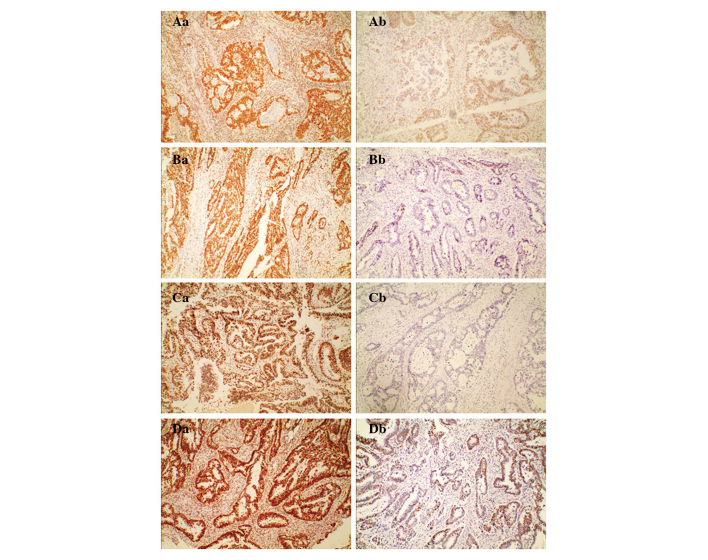
Immunohistochemical staining showing normal and aberrant MMRP expression (magnification, ×100). (A) hMLH1, (B) hMSH2, (C) hPMS2 and (D) hMSH6. (Aa-Da) Normal immunohistochemical staining of (Aa) hMLH1, (Ba) hMSH2, (Ca) hPMS2 and (Da) hMSH6. Normal nuclear staining of the MMRPs can be observed not only in stromal cells, but also in epithelial tumor cells, showing a brownish accumulation of dye in the nucleus. (Ab-Db) Aberrant staining of (Aa) hMLH1, (Ba) hMSH2, (Ca) hPMS2 and (Da) hMSH6. Aberrant nuclear staining of the MMRPs can only be observed in stromal cells, not in epithelial tumor cells. MMRP, mismatch repair protein; hMLH1, human mutL homolog 1; hMSH, human mutS homolog; hPMS2, human postmeiotic segregation increased 2.

In 404 cases with sporadic colorectal cancer, 110 (27.23%) patients showed aberrant nuclear staining for MMRP. For patients with only one type of aberrant expression, hMLH1 expression was absent in 17 cases, hMSH2 in 9 cases, hPSM2 in 7 cases and hMSH6 in 5 cases. In patients with more than one type of aberrant MMRP expression, the protein expression of hMLH1/hMSH2/hPSM2/hMSH6 was absent in 3 cases, hMLH1/hMSH2/hMSH6 in 3 cases, hMSH2/hPSM2/hMSH6 in 4 cases, hMLH1/hPSM2 in 29 cases, hMSH2/hMSH6 in 17 cases, hMLH1/hMSH2 in 7 cases, hMLH1/hMSH6 in 5 cases and hMSH2/hPSM2 in 4 cases. The highest frequency of aberrant MMRP expression was for hMLH1/hPSM2 (Fig. 1).

### Univariate analysis between MMRP expression and clinicopathological parameters

Using univariate analysis, aberrant MMRP expression in colorectal cancer was found to be closely associated with tumor location, histological type, BMI and family history of cancer, and this was statistically significant (P<0.05; Table I).

### Multivariate analysis between MMRP expression and clinicopathological parameters

Using logistic regression analysis, independent risk factors for aberrant MMR were identified; these included tumor location, histological type, BMI and family history of cancer (P<0.05; Table II).

### Survival analysis of normal and aberrant MMRP expression groups

**Figure 2:**
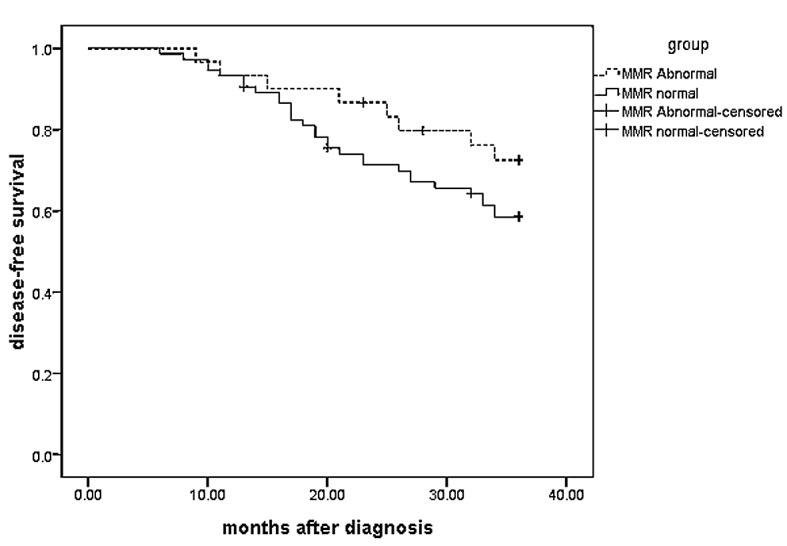
Survival comparison between normal and aberrant MMRP expression groups. The three-year DFS rate was 58.65%, of which 20 cases showed aberrant expression of MMRP. The aberrant MMRP expression group had a three-year DFS rate of 66.67%; this was higher than that of the normal group, which had a three-year DFS rate of 55.41%, but no statistical difference was observed (P>0.05). Censored values were calculated due to events such as unrelated mortality. MMRP, mismatch repair protein; DFS, disease-free survival.

A total of 104 cases with stage III colorectal cancer were randomly selected from 404 cases and followed up for >3 years. A total of 5 cases were lost midway through the follow-up: Three patients succumbed (two due to other diseases and one due to a traffic accident), one patient moved abroad, leading to a loss of contact, and one patient quit the study midway due to mental disease. In total, 74 cases had normal MMRP expression, while 30 cases exhibited aberrant MMR expression. The three-year overall survival was 70.19%, and 31 cases succumbed from distant metastasis or local recurrence. However, the three-year DFS rate was 58.65%, of which 20 cases showed aberrant MMRP expression. The Kaplan-Meier survival curve showed that the aberrant expression group had a three-year DFS rate of 66.67%, which was higher than the three-year DFS rate of the normal group (55.41%). However, no statistical difference was found using the Log-Rank test (P>0.05; Fig. 2).

## Discussion

To date, a number of previous studies have confirmed that immunohistochemistry is a reliable method for MMR gene analysis (23–26). The method has been utilized in the majority of hospitals and research institutions, and has been shown to be cost effective, stable and with a high sensitivity (77–100%) and specificity (98–100%) (23,24). As a result, the immunohistochemical method has been suggested as the preferred method for MMR gene mutation analysis (25,26). The immunohistochemistry PV-9000 two-step method is an enzymatic biotin method. Monovalent Fab fragments of second antibody molecules polymerize with enzymes instead of the traditional method of secondary and tertiary antibodies. Consequently, the antigen-antibody binding signal is directly amplified. Compared with the traditional streptavidin-peroxidase three-step method, the PV-9000 two-step method is simple, fast and sensitive. In addition, it avoids background staining due to a lack of biotin. Thus, the immunohistochemistry PV-9000 two-step method is often used in clinical practice. In the present study, the immunohistochemistry PV-9000 two-step method was performed to measure the expression levels of hMLH1 hMSH2, hPMS2 and hMSH6 in 404 postoperative pathological specimens.

Numerous studies have investigated MLH1 and MSH2 expression (27,28); however, fewer studies have investigated MSH6 and PSM2. It has been shown that the rate of aberrant MLH1 expression is higher than that of MSH2 (23). This may be due to the inactivation of the MLH1 gene in somatic cells (29). CpG islands within the MLH1 gene promoter region are hypermethylated. This methylation causes barriers against gene transcription and translation, resulting in aberrant MLH1 expression. Aberrant MLH1/PSM2 expression is the most common type of aberrant MMR gene expression due to the high frequency of MLH1 methylation and easy heterodimer formation. Correspondingly, aberrant PSM2 expression becomes relatively higher (30,31). In the present study, only one type of aberrant MMRP expression was observed in 38 cases (9.4%). Aberrant expression of hMLH1/hPSM2 showed the highest rate (26.36%), while the rate of aberrant hMSH2/hMSH6 expression was the second highest (15.45%). The results from this study were consistent with those from a previous study by Molaei *et al* (32).

A number of previous studies (33,34) have demonstrated that aberrant MMR is associated with certain clinicopathological features. This association has an important role in the clinical diagnosis and treatment of colorectal cancer. In the present study, cases where the tumor was in the right hemicolon or the tissue type was mucus gland or signet ring cell carcinoma were found to have a higher incidence of aberrant MMRP expression, which is consistent with the results from previous studies (23,35). This may be due to the fact that aberrant MMRP expression is closely associated with MSI-H. The clinicopathological features of right hemicolon tumors or mucinous adenocarcinomas include MSI-H (36–38). In the present study, no difference was observed between rectal and left hemicolon tumors with regard to MMRP expression, suggesting that the aberrant MMR expression pathways exhibit consistency. However, a statistical difference was observed between left and right hemicolon carcinomas, suggesting that a higher incidence of gene promoter hypermethylation may occur in the right hemicolon tissues, leading to the occurrence of MSI.

In the present study it was demonstrated that the rate of aberrant MMRP expression was not associated with age at diagnosis, gender, nationality, anemia, tumor size or TNM staging (P>0.05). The association between anemia and MMRP expression has, to this date, been unclear. The rate of aberrant MMRP expression in the anemia group (23.15%) was lower than that in the normal hemoglobin group (28.72%); however, this difference was not statistically significant (P>0.05). Tumors with aberrant MMRP expression were mostly located in right hemicolon, the clinical manifestations of which showed a higher risk of anemia. Further studies are required to elucidate the specific association between these factors.

With improvements in living standards, dietary structure has also been changing. The dietary habit of consuming more meat and less fiber has caused an increasing incidence of overweight and obese individuals. An increasing number of studies are focusing on the association between BMI and colorectal cancer. Several studies have shown a close correlation between increasing BMI and risk factors of colorectal cancer (39,40). However, with the exception of the study by van Duijnhoven *et al* (41), which described certain aspects of the association between BMI and MMR gene expression, studies focusing on the association between BMI and MMR gene expression are relatively rare. In the present study, increasing BMI was significantly correlated with aberrant MMRP expression. In the study by Botma *et al* (42), it was revealed that BMI had a close correlation with colorectal adenomas; however, the study subjects were all male. In the study by Win *et al* (43), manifested BMI was reported to be a potential risk factor for individuals in early adulthood carrying MMR gene mutations. Therefore, previous study results suggest that MMR gene mutation occurs in the early pathogenetic stage of colorectal cancer. Being overweight or obese may be independent risk factors of aberrant MMR gene expression. However, further studies are required to investigate the underlying mechanism, as this has yet to be elucidated.

Studies investigating whether the pathogenesis of sporadic colorectal cancer in patients with a tumor familial history is the same as that of HNPCC are rare. Germline MMR gene mutations have been identified as the molecular genetic basis underlying HNPCC. By contrast, mutations in the adenomatous polyposis coli gene are believed to comprise the molecular genetic basis underlying familial adenomatous polyposis and the majority of sporadic colorectal cancer cases. Sporadic colorectal cancer additionally exhibits a polygenic and multi-stage process of tumor formation, which includes activating mutations in adenoma-carcinoma sequences in oncogenes and inactivating mutations in tumor suppressor genes (44,45). In the present study, the rate of aberrant MMRP expression in the group with a family history of cancer (36.27%) was higher than that in the group without a family history of cancer (24.17%), with a statistically significant difference (P<0.05). Therefore, cancer family history was correlated with aberrant MMR expression.

A number of studies have revealed that patients with a positive MSI in colorectal cancer show a more favorable prognosis (46,47); however, the mechanism associated with this remains unclear. Popat *et al* (18) reported that, although colorectal cancer with MSI-H had numerous features associated with a poor prognosis, MSI-H was also associated with a relatively good prognosis due to increased inflammatory cell infiltration. In addition, Sargent *et al* (20) revealed that cancer with MSI-H was not sensitive to 5-FU-based chemotherapy. However, as to whether it is associated with MMR gene mutations, a number of studies (48,49) have produced affirmative results. In the present study, 104 patients with stage III colorectal cancer were followed up for >3 years. Survival analysis showed that the three-year DFS of the aberrant MMRP expression group was higher than that of the normal expression group. However, no statistically significant difference was identified between the groups (P>0.05). This may be due to the fact that patients with aberrant MMRP expression had a higher MSI, which, according to the above studies, was a good prognostic factor.

In conclusion, the immunohistochemistry PV-9000 two-step method can be feasibly used to detect the MMRP expression level in sporadic colorectal cancer. MMRP expression is closely associated with tumor location, histological type, differentiation degree, BMI and a family history of cancer, respectively. MMRP expression level may be a promising prognostic factor. Therefore, MMR plays a significant role in the occurrence and development of colorectal cancer; further studies are required to explore its detailed mechanism.

## Figures and Tables

**Table I:** Univariate analysis between MMRP expression and clinicopathological parameters.

Clinicopathological index	Normal MMRP, n=294	Aberrant MMRP, n=110	Total, n=404	χ^2^	P-value
Age (years)				0.274	0.601
<50	88	30	118		
≥50	206	80	286		
Gender				0.335	0.563
Male	167	66	233		
Female	127	44	171		
Nationality				3.907	0.272
Han	219	73	292		
Uyghur	38	16	54		
Hui	21	12	33		
Others	14	9	25		
BMI (kg/m^2^)				7.911	0.048
<18.5	20	4	24		
18.5–23.99	133	37	170		
24–27.99	107	49	156		
≥28	34	20	54		
Anemia				1.238	0.266
Yes	83	25	108		
No	211	85	296		
Tumor size (cm)				1.258	0.533
<4	68	25	93		
4–6	156	53	209		
≥6	70	32	102		
Tissue type				7.226	0.007
Glandular	244	78	322		
Mucous gland/signet cell	50	32	82		
Differentiation degree				8.119	0.004
Well/moderately	200	58	258		
Poorly	94	52	146		
Tumor general type				0.257	0.880
Ulcerative	196	76	272		
Mass	85	29	114		
Infiltrative	13	5	18		
TNM staging				1.061	0.786
I	21	8	29		
II	104	33	137		
III	144	59	203		
IV	25	10	35		
Tumor location				11.607	0.003
Rectal	159	46	203		
Left hemicolon	86	28	114		
Right hemicolon	51	36	87		
Familial cancer history				7.510	0.023
Colorectal cancer	20	16	36		
Others	45	21	66		
No	229	73	302		

MMRP, mismatch repair protein; BMI, body mass index.

**Table II:** Multivariate analysis results between MMRP expression and clinicopathological parameters.

			95% confidence interval		
				
Variable	B-value	OR	Lower bound	Upper bound	P-value
BMI (kg/m^2^)					
≤24 vs*.* ≥24	0.341	0.711	0.529	0.956	0.024
Histological type					
Glandular vs*.* mucous gland/signet cell	0.609	1.838	1.072	3.152	0.027
Tumor location					
Left hemicolon/rectal vs*.* right hemicolon	0.761	0.467	0.278	0.784	0.004
Family history of cancer					
Positive vs*.* negative	0.413	1.511	1.075	2.124	0.017

MMRP, mismatch repair protein; BMI, body mass index; OR, odds ratio.

## References

[1] Adib SM, Tabbal N, Hamadeh R, Ammar W (2013). Geographic epidemiology in a small area: cancer incidence in Baakline, Lebanon, 2000–2008. East Mediterr Health J.

[2] Van Engeland M, Derks S, Smits KM (2011). Colorectal cancer epigenetics: complex simplicity. J Clin Oncol.

[3] Blokhuis MM, Pietersen GE, Goldberg PA (2010). Lynch syndrome: the influence of environmental factors on extracolonic cancer risk in hMLH1 c. C1528T mutation carriers and their mutation-negative sisters. Fam Cancer.

[4] Fishel R, Lescoe MK, Rao MR (1993). The human mutator gene homolog MSH2 and its association with hereditary nonpolyposis colon cancer. Cell.

[5] Aaltonen LA, Salovaara R, Kristo P (1998). Incidence of hereditary nonpolyposis colorectal cancer and the feasibility of molecular screening for the disease. N Engl J Med.

[6] Mureşan F, Simescu R, Domşa I (2011). Immunohistochemical screening of hMLH1 and hMSH2 gene mutations in patients diagnosed with colorectal cancer and microsatellite instability suspicion. Chirurgia (Bucur).

[7] Wei W, Liu F, Liu L (2011). Distinct mutations in MLH1 and MSH2 genes in hereditary non-polyposis colorectal cancer (HNPCC) families from China. BMB Rep.

[8] Rodriguez-Bigas MA, Boland CR, Hamilton SR A National Cancer Institute Workshop on Hereditary Nonpolyposis Colorectal Cancer Syndrome: meeting highlights and Bethesda guidelines. J Natl Cancer Inst.

[9] Lynch HT, Smyrk T (1996). Hereditary nonpolyposis colorectal cancer (Lynch syndrome). An updated review. Cancer.

[10] Win Aung Ko, Young Joanne P (2012). Colorectal and other cancer risks for carriers and noncarriers from families with a DNA mismatch repair gene mutation: a prospective cohort study. J Clin Oncol.

[11] Wada-Hiraike O, Yano T, Nei T (2005). The DNA mismatch repair gene hMSH2 is a potent coactivator of oestrogen receptor alpha. Br J Cancer.

[12] Park IJ, Kim HC, Kim JS (2005). Correlation between hhMLH1/hhMSH2 and p53 protein expression in sporadic colorectal cancer. Hepatogastroenterology.

[13] Vreeswijk MP, van der Klift HM (2012). Analysis and interpretation of RNA splicing alterations in genes involved in genetic disorders. Methods Mol Biol.

[14] Sun ZQ, Yu XB, Wang HJ (2014). Relationship between Aberrant Expression of hMSH2 and prognosis in patients with sporadic colorectal cancer. Canc Cell Rec.

[15] Belcheva A, Irrazabal T, Robertson SJ (2014). Gut microbial metabolism drives transformation of msh2-deficient colon epithelial cells. Cell.

[16] Cunningham JM, Kim CY, Chirstensen ER (2001). The frequency of hereditary defective mismatch repair in a prospective series of unselected colorectal carcinomas. Am J Hum Genet.

[17] Ionov Y, Peinado MA, Malkhosyan S (1993). Ubiquitous somatic mutations in simple repeated sequences reveal a new mechanism for colonic carcinogenesis. Nature.

[18] Popat S, Hubner R, Houlston RS (2005). Systematic review of microsatellite instability and colorectal cancer prognosis. J Clin Oncol.

[19] Ribic CM, Sargent DJ, Moore MJ (2003). Tumor microsatellite-instability status as a predictor of benefit from fluorouracil-based adjuvant chemotherapy for colon cancer. N Engl J Med.

[20] Sargent DJ, Marsoni S, Monges G (2010). Defective mismatch repair as a predictive marker for lack of efficacy of fluorouracil-based adjuvant therapy in colon cancer. J Clin Oncol.

[21] Holzhüter J, Rösch T, Block A (2013). A 44-year-old woman with hereditary nonpolyposis colon carcinoma: screening examinations for non-colonic tumors. Internist (Berl).

[22] Plevová P, Krepelová A, Papezová M (2004). Immunohistochemical detection of the hMLH1 and hMSH2 proteins in hereditary non-polyposis colon cancer and sporadic colon cancer. Neoplasma.

[23] Lindor NM, Burgart LJ, Leontovich O (2002). Immunohistochemistry versus microsatellite instability testing in phenotyping colorectal tumors. J Clin Oncol.

[24] Ruszkiewicz A, Bennett G, Moore J (2002). Correlation of mismatch repair genes immunohistochemistry and microsatellite instability status in HNPCC-associated tumours. Pathology.

[25] Shia J, Klimstra DS, Nafa K (2005). Value of immunohistochemical detection of DNA mismatch repair proteins in predicting germline mutation in hereditary colorectal neoplasms. Am J Surg Pathol.

[26] Marcus VA, Madlensky L, Gryfe R (1999). Immunohistochemistry for hMLH1 and hMSH2: a practical test for DNA mismatch repair-deficient tumors. Am J Surg Pathol.

[27] Joost P, Veurink N, Holck S (2014). Heterogenous mismatch-repair status in colorectal cancer. Diagn Pathol.

[28] Haghighi MM, Aghagolzadeh P, Zadeh SM (2014). Telomere shortening: a biological marker of sporadic colorectal cancer with normal expression of p53 and mismatch repair proteins. Genet Test Mol Biomarkers.

[29] Hawkins NJ, Ward RL (2001). Sporadic colorectal cancers with microsatellite instability and their possible origin in hyperplastic polyps and serrated adenomas. J Natl Cancer Inst.

[30] Kupčinskaitė-Noreikienė R, Skiecevičienė J, Jonaitis L (2013). CpG island methylation of the MLH1, MGMT, DAPK, and CASP8 genes in cancerous and adjacent noncancerous stomach tissues. Medicina (Kaunas).

[31] Gomes A, Reis-Silva M, Alarcão A (2014). Promoter hypermethylation of DNA repair genes MLH1 and MSH2 in adenocarcinomas and squamous cell carcinomas of the lung. Rev Port Pneumol.

[32] Molaei M, Mansoori BK, Ghiasi S (2010). Colorectal cancer in Iran: immunohistochemical profiles of four mismatch repair proteins. Int J Colorectal Dis.

[33] Bellizzi AM, Frankel WL (2009). Colorectal cancer due to deficiency in DNA mismatch repair function: a review. Adv Anat Pathol.

[34] Valle L, Perea J, Carbonell P (2007). Clinicopathologic and pedigree differences in amsterdam I-positive hereditary nonpolyposis colorectal cancer families according to tumor microsatellite instability status. J Clin Oncol.

[35] Jass JR (2004). HNPCC and sporadic MSI-H colorectal cancer: a review of the morphological similarities and differences. Fam Cancer.

[36] Raskin GA, Ianus GA, Kornilov AV (2014). Immunohistochemical examination of MSH2, PMS2, MLH1, MSH6 compared with the analysis of microsatellite instability in colon adenocarcinoma. Vopr Onkol.

[37] Kim JH, Kang GH (2014). Molecular and prognostic heterogeneity of microsatellite-unstable colorectal cancer. World J Gastroenterol.

[38] Goldstein J, Tran B, Ensor J (2014). Multicenter retrospective analysis of metastatic colorectal cancer (CRC) with high-level microsatellite instability (MSI-H). Ann Oncol.

[39] Slattery ML, Levin TR, Ma K (2003). Family history and colorectal cancer: predictors of risk. Cancer Causes Control.

[40] Campbell PT, Cotterchio M, Dicks E (2007). Excess body weight and colorectal cancer risk in Canada: associations in subgroups of clinically defined familial risk of cancer. Cancer Epidemiol Biomarkers Prev.

[41] van Duijnhoven FJ, Botma A, Winkels R (2013). Do lifestyle factors influence colorectal cancer risk in Lynch syndrome?. Fam Cancer.

[42] Botma A, Nagengast FM, Braem MG (2010). Body mass index increases risk of colorectal adenomas in men with Lynch syndrome: the GEOLynch cohort study. J Clin Oncol.

[43] Win AK, Dowty JG, English DR (2011). Body mass index in early adulthood and colorectal cancer risk for carriers and non-carriers of germline mutations in DNA mismatch repair genes. Br J Cancer.

[44] Sánchez-de-Abajo A, de la Hoya M, van Puijenbroek M (2007). Molecular analysis of colorectal cancer tumors from patients with mismatch repair proficient hereditary nonpolyposis colorectal cancer suggests novel carcinogenic pathways. Clin Cancer Res.

[45] Karoui M, Tresallet C, Brouquet A (2007). Colorectal carcinogenesis. 1. Hereditary predisposition and colorectal cancer. J Chir (Paris).

[46] Bubb VJ, Curtis LJ, Cunningham C (1996). Microsatellite instability and the role of hhMSH2 in sporadic colorectal cancer. Oncogene.

[47] Gryfe R, Kim H, Hsieh ET (2000). Tumor microsatellite instability and clinical outcome in young patients with colorectal cancer. N Engl J Med.

[48] Sankila R, Aaltonen LA, Järvinen HJ, Mecklin JP (1996). Better survival rates in patients with hMLH1-associated hereditary colorectal cancer. Gastroenterology.

[49] Heinimann K, Scott RJ, Buerstedde JM (1999). Influence of selection criteria on mutation detection in patients with hereditary nonpolyposis colorectal cancer. Cancer.

